# Integrated cardiac, endocrine, and genetic assessment in adolescent basketball players

**DOI:** 10.3389/fendo.2026.1851858

**Published:** 2026-07-20

**Authors:** Katerina Gregorova, Lukas Plachy, Vaclav Chaloupecky, Olena Iurchenko, Klara Maliska Maratova, Aneta Kodytkova, Martin Matoulek, Jan Lebl, Viktor Tomek, Zdenek Sumnik, Petra Dusatkova, Stepanka Pruhova

**Affiliations:** 1 Department of Pediatrics, 2nd Faculty of Medicine, Charles University, Prague, Czechia; 2 Department of Pediatrics, University Hospital Motol and Homolka, Prague, Czechia; 3 Children’s Heart Centre, 2nd Faculty of Medicine, Charles University, Prague, Czechia; 4 Children’s Heart Centre, University Hospital Motol and Homolka, Prague, Czechia; 5 Department of Endocrinology and Metabolism, 1st Faculty of Medicine, Charles University, Prague, Czechia; 6 Department of Endocrinology and Metabolism, General University Hospital, Prague, Czechia

**Keywords:** basketball, dysmorphia, echocardiogram (ECG), genetics, tall stature

## Abstract

**Introduction:**

Athletes pursuing a professional basketball career are routinely evaluated by sports physicians using laboratory testing, electrocardiography, and exercise stress assessment. However, such screening may not detect certain cardiovascular or connective tissue disorders. This limitation is particularly relevant in basketball, where tall stature is advantageous but may also signal underlying conditions such as cardiomyopathies, which can predispose athletes to life-threatening events during intense physical exertion, including sudden cardiac death. This study aimed to investigate the phenotypic, endocrine, cardiac, and genetic characteristics of adolescent basketball players to identify possible causes of tall stature and associated health risks.

**Methods:**

We examined 55 youth basketball players (30 females, median age 17 years and height +2.4 standard deviations). Each underwent detailed endocrine and anthropometric examinations, echocardiography, and genetic testing (number of X and Y chromosomes, *SHOX* gene dosage analysis, and a custom next-generation sequencing panel of 786 genes related to growth).

**Results:**

Echocardiographic abnormalities were observed in 4/55 (7%) athletes, while an additional 5/55 (9%) probands displayed echocardiographic findings resembling physiological adaptation to extensive physical activities. Clinically relevant dysmorphic features were identified in 26/55 (47%), and mild dysmorphia was present in 21/55 (38%) participants. No endocrine dysfunctions were detected. One proband (1.8%) was diagnosed with a monogenic cause of tall stature carrying a pathogenic variant in the *FBN1* gene, causing Marfan syndrome.

**Discussion:**

Echocardiographic abnormalities were observed in a small proportion of probands, as were monogenic causes of tall stature. However, targeted evaluation, including echocardiography, anthropometry, and genetic testing, should be considered in selected individuals.

## Introduction

Tall stature is a very important attribute for basketball players. However, it can be associated with severe health risks that should be considered, including endocrine (1, 2) (e.g., growth hormone overproduction), cardiovascular (3, 4) (e.g., aortic aneurysms, valve diseases), musculoskeletal (5) (e.g., scoliosis, joint diseases) and oncological (6). Moreover, people with tall stature have a shorter life span compared to their peers (7). Importantly, the potential health complications associated with tall stature can cause severe or fatal health conditions during sports performance: e.g., sudden cardiac death (SCD) (8). As repeatedly shown by Maron et al. (8–10), basketball players have the highest detection rate of SCD compared with other athletes (football, American football, swimming). In addition, the overall annual incidence of sudden cardiac death (SCD) among college athletes in the United States was 1 in 63,682 (9), while among male basketball players it was 1 in 9,000. Furthermore, an incidence of SCD of 1 in 5,300 was reported among Black male basketball players (8).

Currently, all professional athletes in the Czech Republic, including basketball players, are monitored by a sports doctor who performs routine laboratory tests, ECG (electrocardiography), and exercise stress tests. However, the diagnostic value of these investigations could be limited (11). On the other hand, an echocardiogram (ECHO) is not mandatory as a first-line investigation, which is consistent with European (12) and United States guidelines (13). Therefore, cardiovascular conditions could remain undiagnosed in these athletes, as it was previously shown in volleyball and female basketball players (14, 15). But considering the possibility of hidden echocardiographic pathology (16), the National Basketball Association (NBA) decided already in 2006 to screen its players before entering the NBA via ECHO to uncover possible hidden pathological conditions (17). However, two decades later, the official international guidelines (12, 13) remain silent about such recommendations.

With broader possibilities of genetic testing, there is a question of whether athletes with tall stature could benefit from genetic testing in order to reveal their possible hidden health risks. There were a few studies focused on the genetic investigation of athletes with tall stature. High polygenic score quantifying small effects from many common genetic variants showed correlation with the phenotype of an extremely tall basketball player measuring 2.29 meters (18). Of the 90 United States Volleyball National Team members, genetic testing was performed on three with dilated sinuses of Valsalva, and one of these players was found to have a pathogenic variant in the *FBN1* gene (14). To the best of our knowledge, there was no study that performed comprehensive genetic testing on a larger group of athletes with tall stature in order to find monogenic causes of their phenotype. As we showed in our previous works (19, 20), hidden genetic causes might be found among persons with tall stature, although their phenotype can be subtle.

Our study aimed to examine youth basketball players to reveal possible endocrine and monogenic causes of tall stature, as well as dysmorphic features and cardiological alterations, and to compare these findings between groups of basketball players with and without tall stature.

## Materials and methods

### Study settings and inclusion criteria

**Figure 1:**
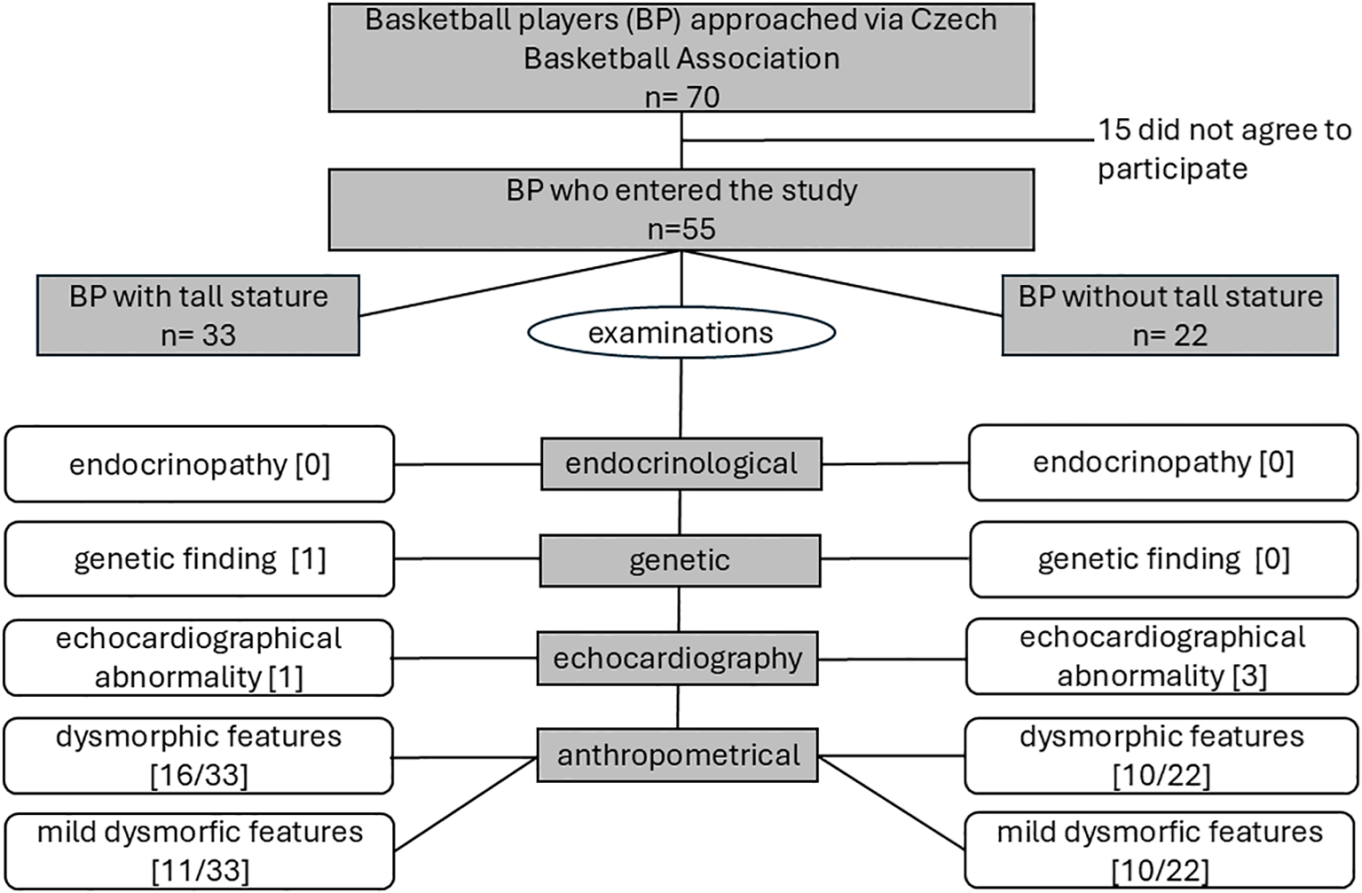
Flowchart of the study. BP, basketball players; SD, standard deviation.

The study was voluntary. The Czech Basketball Association offered participation in the study to players heading toward or already pursuing a professional basketball career, following these inclusion criteria: age > 10 years old, basketball practice at least six hours per week. Those who agreed with the study, fulfilled the inclusion criteria, and whose parents or legal guardians signed written consent, entered the study. The participants were divided into two groups according to their height: persons with height > 2 standard deviations (SD), i.e., the group with tall stature, and persons with height < 2 SD, i.e., the group without tall stature. The detailed flowchart of the study is shown in **Figure 1**. The study was conducted in accordance with the Declaration of Helsinki. It was approved by the Ethical Committee of the University Hospital Motol and the Second Faculty of Medicine, Charles University, on 17^th^ June 2020.

### Endocrine and anthropometrical examination

Endocrine and anthropometric investigations of the basketball players were performed using the protocol reported in our previous studies (19, 20). Briefly, all study participants were examined by a pediatric endocrinologist. Hormonal testing was performed mainly to exclude hyperthyroidism and growth hormone excess. Bone age was evaluated using the Tanner-Whitehouse method (21). Consequently, all study participants were examined by a clinical anthropologist trained in the recognition of syndromic clinical signs. The dysmorphic features were assessed using a scoring system (**Supplementary Table 1**) published in our previous work (20) dividing participants into three groups: no dysmorphia, mild dysmorphia, and clinically relevant dysmorphia based on their phenotypic features.

### Echocardiography

All the study participants underwent an ECHO examination by a pediatric cardiologist. ECHO data were analyzed in the laboratory using GE Vivid E95 ultrasound and the EchoPac software. The ECHO protocol included: two-dimensional grey scale loops of the parasternal long-axis view (LAX), parasternal short-axis view (SAX), suprasternal view, apical four-chamber and two-chamber views. The left ventricle (LV) systolic function was assessed by calculating ejection and shortening fractions from LAX and SAX M-mode. Subsequent measurements included the E/A ratio, transmitral E deceleration time, isovolumic relaxation time, early diastolic mitral annular Tissue Doppler Imaging (e′), and left atrium (LA) strain analysis for assessing LV diastolic function. The Pulsed Doppler was used for the right and left ventricular outflow and inflow tracts, qualitative assessment of mitral, tricuspid, pulmonary, and aortic valve regurgitation. Finally, the area of left and right atria, diameters of mitral, tricuspid, pulmonary, and aortic valve annuli, diameter of the aortic root, ascending aorta, aortic arch, and descending aorta were assessed. Z scores compared to body surface area for all measurements using the worldwide accepted normative values (22, 23) were used. The ECHO findings were subsequently divided into three groups: potentially pathological findings, borderline findings, and findings of physiological adaptation to significant sport activity. Borderline and potentially pathological findings were reanalyzed and compared to reference values for the athletic population (24). Two cardiac cycles were recorded in each view, along with simultaneous ECG tracing to allow for identification of QRS onset with 4 MHz transducers with a minimal frame rate of 50/s. The echocardiographic examination was performed without knowledge of the anthropometric and genetic findings.

### Genetic examination and bioinformatic evaluation

Study participants were tested for chromosome X or Y aneuploidy and for the dosage of the *SHOX* gene using the Multiplex Ligation Probe-dependent Amplification (MLPA kits P095 and P018, MRC Holland, Amsterdam, Netherlands). In case of a negative result, the DNA of each proband was analyzed using a custom targeted next-generation sequencing panel (Agilent Technologies, Santa Clara, USA) of 786 genes associated with overgrowth or involved in the development and regulation of the pituitary and growth plate, as reported previously, as well as a bioinformatic pipeline (19). Segregation of observed single-nucleotide variants of interest was confirmed in available parents using Sanger sequencing (25). The American College of Medical Genetics and Genomics standards and guidelines (26, 27) were used for the final evaluation and classification of the detected variants into five groups: pathogenic, likely pathogenic, benign, likely benign, or variants of uncertain significance (VUS).

### Statistical analysis

Statistical comparisons of the results from the groups with height > 2 SD and < 2 SD were performed using the Mann-Whitney test (continuous variables) and the chi–square test of independence (categorical variables). P values < 0.05 were considered statistically significant. The data is available upon request, as it cannot be published due to privacy concerns or ethical restrictions.

## Results

In total, 55 youth athletes (30 girls) were enrolled in the study. The median age was 17 years (interquartile range 16–19 years), body height 2.4 SD (1.4 - 2.6 SD), sitting height to subischial length (SHSI) ratio -0.8 SD (-1.7 to -0.1 SD) (**Table 1**). There were 33 basketball players in the group with tall stature (height > 2 SD (19 girls)) and 22 players in the group without tall stature (height < 2 SD (11 girls)). Significantly higher birth length was observed in participants from the group with tall stature compared to those without tall stature (**Table 2**). Four persons were already followed up before entering the study for a hemodynamically insignificant cardiovascular condition (foramen ovale apertum (in two), mild mitral regurgitation, insignificant valve murmur), and two had a history of mild patellar dysplasia and clinically asymptomatic sacralization of the sixth lumbar vertebrae. In addition, one proband was followed up for an autoimmune thyroiditis on levothyroxine supplementation with a stable hormonal status (**Supplementary Table 2**).

**Table 1:** Clinical description and overall results of the cohort.

Characteristics	n=55
Gender (female/male)	30/25
Age (years)	17 (16–19)
Birth length (SD) *	0.5 (-0.2 - 1.1)
Birth weight (SD) *	1.1 (0.3 - 1.7)
Maximal height (SD) *	2.4 (1.4 - 2.9)
Sitting height to subischial length (SD) *	-0.8 (-1.7 - -0.1)
Clinically relevant dysmorphia (%)	47
Mild dysmorphia (%)	38
Mid-parental height (SD) *	1.4 (0.6 - 1.8)
Detected genetic cause of tall stature (%)	2
VUS in the genes associated with tall stature (%)	18
ECHO positive finding (%)	7
Positive family history of cardiovascular diseases (%)	49

* Data displayed as SD with interquartile range.

ECHO, echocardiogram; SD, standard deviation; VUS, variant of uncertain significance.

**Table 2:** Comparison of the participants with tall stature and those without tall stature.

Characteristics	Tall stature height > 2 SD (n=33)	No tall stature height < 2 SD (n=22)	P-value
Gender (female/male)	19/14	11/11	0.778
Age (years)	17.0 (16.0-19.0)	16.0 (16.0-17.0)	0.992
Birth length (SD) *	1.1 (0.1-1.4)	0.1 (-0.2-0.5)	0.020
Birth weight (SD) *	1.6 (0.3-1.9)	0.7 (0.3-1.1)	0.240
Maximal height (SD) *	2.7 (2.4-3.1)	1.1 (0.7-1.5)	<0.0001
Sitting height to subischial length (SD) *	-1.2 (-2.1 - -0.2)	-0.4 (-1.0-0.0)	0.006
Clinically relevant dysmorphia (%)	48	45	0.823
Mild dysmorphia (%)	33	45	0.365
Mid-parental height (SD) *	1.4 (0.7-2.0)	1.2 (0.5-1.6)	0.347
Detected genetic cause of tall stature (%)	3	0	NA
VUS in the genes associated with tall stature (%)	24	9	0.284
ECHO abnormal finding (%)	3	14	0.290
Positive family history of cardiovascular diseases (%)	58	36	0.475

*Data displayed as SD with interquartile range.

NA, not available; SD, standard deviation.

Potentially pathological or borderline cardiovascular findings (newly diagnosed besides the already known conditions) were observed in four out of 55 (7%) basketball players. Specifically, a potentially pathological finding (hypertrophy of the interventricular septum) was observed in one proband, and borderline findings were detected in three probands (dilatation of the ascending aorta in two, dilatation of the aortic root in one). Furthermore, five probands were displaying physiological adaptation to extensive sport activity (left ventricular hypertrophy, left atrium enlargement) (**Supplementary Tables 2**, **3**). All basketball players had normal diastolic function and normal function of the right ventricle.

No hormonal abnormalities were detected. Clinically relevant dysmorphic features (more than 4 points based on the previously published scoring system) were described in 26/55 (47%) participants by clinical anthropologists. In addition, 21/55 (38%) basketball players showed mild dysmorphia (1–3 points). For details, see **Supplementary Tables 1**, **2**. There were 20/55 (36%) participants with marfanoid features, including arachnodactyly (positive thumb and wrist sign), chest asymmetry/deformity, high-arched palate, or joint hypermobility. Comparison of anthropometrical characteristics revealed that probands with tall stature exhibited significantly greater body disproportionality compared to those without tall stature (**Table 2**).

The genetic cause of tall stature was identified in only one participant carrying a *de novo* heterozygous pathogenic variant in the *FBN1* gene, causing Marfan syndrome. This patient had mild dysmorphic features (pectus excavatum, striae) but no pathological findings on ECHO. VUS were identified in an additional 10 probands (**Supplementary Table 2**).

## Discussion

To the best of our knowledge, this is the first study comprehensively evaluating clinical, anthropometric, genetic, and cardiovascular characteristics in youth basketball players.

One subject was found to have a previously undiagnosed potentially pathological abnormality (hypertrophy of the interventricular septum) using ECHO. While five of the ECHO findings in the studied cohort could be associated with typical athletic heart adaptations (left ventricular hypertrophy, left atrium enlargement), three detected features, like dilatation of the aortic root and ascending aorta (probands no. 30, 37, 52, **Supplementary Tables 2**, **3**), may indicate underlying connective tissue disorders and a predisposition to aortic dissection. But in case of the respective probands, none of the aortic dilatations was currently too severe, so the cardiologists indicated these probands for further follow-up after this study but did not recommend restriction from sports (28, 29). Our findings align with previous research in adult professional athletes (17) showing a predominance of athletic heart presentations (left ventricular hypertrophy found in 27% of the participants) but also identified undetected cardiomyopathies or aortic abnormalities associated with SCD. Importantly, none of these significant features are typically revealed through ECG alone. Additionally, a study in female basketball players has demonstrated a high prevalence of mitral valve incompetence (37%), particularly among those with marfanoid features (15). Only longitudinal follow-up can clarify whether currently designated normal variations in young athletes may eventually predispose some of them to cardiovascular complications in adulthood. Our findings underscore the need to evaluate the potential long-term cardiac impact of sustained physical activity in adolescents with subtle ECHO abnormalities.

Clinically relevant dysmorphic features were described in 47% subjects, and specifically marfanoid characteristics were described in 36% of participants. These results are in line with our previous study focused on children and adolescents with non-familial tall stature (nFTS), showing dysmorphic signs suggestive of connective tissue disorders in 53% (20). In addition, 50% of the probands with potentially pathological or borderline ECHO findings had clinically relevant dysmorphic features, and 25% had mild dysmorphic features according to the scoring system of the dysmorphic features. The scoring system was designed to improve the identification of mild dysmorphic features in individuals who remain at risk for connective tissue disorders and is based on dysmorphic features associated with connective tissue disorders and tall stature. Although the scoring system cannot guarantee its specificity in relation to the phenotype-genotype correlation, it should identify individuals at potential risk for further investigation, as was demonstrated, for example, in a proband in whom a pathogenic variant of the *FBN1* gene was subsequently detected.

The monogenic cause of tall stature was revealed in one study participant (1.8%). This proportion of positive genetic findings was the lowest in comparison to our previous studies focusing on children with familial tall stature (FTS) (19) with a positive detection rate of 32% and with 11% of children with nFTS (20) carrying pathogenic genetic variants. No causal genetic variant was detected in basketball players with a positive family history of tall stature (20/55). The only detected pathogenic variant in participant no. 22 (**Supplementary Table 2**) was observed to arise *de novo*, which is in line with the height of his parents (midparental height 0.53 SD). Positive genetic findings were therefore detected in 2.9% (1/35) of basketball players with nFTS, which is lower than 11% detection rate from the nFTS cohort described previously (20). The low prevalence of pathogenic genetic variants is consistent with the origin of the study cohort, which was young, active, healthy basketball players. Low detection rate of genetic findings is also in line with other work done in this field, such as the study by Herrick et al. (14) Ninety volleyball players were screened through ECHO to uncover four athletes with aortic root dilatation. Three of them were subsequently tested via next-generation sequencing, which revealed one patient with the *FBN1* pathogenic gene variant. These four patients with aortic root dilatation did not fulfill the Ghent score (the maximal Ghent score was 4 points for one of the participants) to be clinically diagnosed with Marfan syndrome.

Although phenotypic features associated with connective tissue disorders were relatively common findings among basketball players (36% displayed marfanoid habitus), positive genetic findings are mostly not found by the currently used genetic methods. The reason for that can be hidden monogenic causes in genes leading to marfanoid phenotype, which were not associated with tall stature yet and would require whole exome or genome sequencing. Moreover, the polygenic trait of inheritance can play a role in the low detection rate of monogenic causes, as was shown by Sexton et al. (18) stating that polygenic score can play an important role in extreme phenotypes. Since only growth-related genes were examined in the cohort, the presence of pathogenic variants in any of the genes involved in the development and function of the cardiovascular system cannot be excluded and requires further investigation. Of note, 10 out of 55 study participants carried VUS, three of which were in genes associated with connective tissue disorders, specifically *FBN1*, causing Marfan syndrome (3), *COL1A1* is associated with Ehlers–Danlos syndrome (30) and *TGFB3* is linked to Loeys–Dietz syndrome (31). These basketball players also exhibited phenotypic features suggestive of a possible underlying connective tissue disorder, although some of the phenotypic features were only mild. As we have already presented in our previous work (20),such findings are common among probands with tall stature. However, it is important to note that at the time of the study, these variants were classified as of uncertain significance based on current scientific knowledge, and no clinical conclusions should be drawn based on them.

The Ghent scoring system is typically used to evaluate dysmorphic features exhibiting Marfan-like characteristics and thus the presumed potential risk of connective tissue disorders. However, based on the results of the assessment of dysmorphic features using the Ghent system, a subject with genetically proven Marfan syndrome from this study would not be indicated for genetic testing. It is therefore important to highlight the risk that there may be individuals with undiagnosed connective tissue disorders in professional sports who are at risk of SCD.

Comparison of participants with tall stature (height > 2 SD) and those without tall stature (height < 2 SD) revealed a similar proportion of ECHO abnormalities, dysmorphic features, and genetic findings. Therefore, tall stature was not proven to be the main risk factor of the possible underlying condition. It is important to note that definitive conclusions about the effect of height should not be drawn from this study alone, given its relatively small sample size. Additionally, as we currently are not able to fully decide about the possible impact of some of the ECHO findings of the screened athletes, more prospective studies are needed to evaluate the outcomes on larger populations to be fully able to decide whether to consider or recommend ECHO screening in professional athletes or youth athletes aiming toward a professional sports career to uncover possible hidden cardiac conditions. It is also important to consider the risk-benefit balance of routine ECHO screening in all athletes. Data from a Spanish cohort of 2,617 athletes showed that the cost per detected cardiovascular disease was €3,080, while the cost per cardiovascular disease associated with SCD reached €12,323 (32). These results highlight the significant economic burden that screening represents for healthcare systems and public insurance companies, especially in resource-limited settings. In addition to financial considerations, universal screening strategies may also have significant psychological consequences. Athletes who are disqualified from competitive sports following abnormal findings, particularly in cases of borderline or uncertain clinical significance, may experience considerable psychological distress, identity disruption, and long-term negative effects on mental well-being (33). Therefore, the implementation of large-scale echocardiographic screening programs should be carefully considered in light of their diagnostic yield, economic impact, and potential psychosocial harm. With the global spread of artificial intelligence systems, there is also the possibility of using artificial intelligence to distinguish pathological ECHO findings, which could be beneficial in the future (34).

Strengths of our study include the use of a uniform protocol across all 55 probands, comprehensive genetic testing, echocardiographic assessment performed by an experienced pediatric cardiologist, and detailed phenotypic characterization by a skilled anthropologist. However, we acknowledge that our study has several limitations. The genetic methods used did not cover all possible genetic causes of disorders, such as noncoding variants (except for disruptions in the exon–intron boundaries up to a distance of 10 nucleotides), variants in uncovered regions using selected methods, and variants in the length of repetitive regions. The ECHO evaluation was performed at rest only, and therefore, functional cardiac changes under physical stress were not assessed. Also, the orthopedic diseases were not assessed by an orthopedist during the study, but they were only reported by the parents/probands. Due to the small sample size of the cohort with a limited statistical power to detect differences, no further divisions of the study group based on either dysmorphic, cardiological, or genetic observation were performed. In addition, the study may be biased by the selected group of athletes, as basketball players tend to be taller than other athletes, e. g., soccer players or gymnasts. Thus, it is expected to find more players with marfanoid habitus (associated with tall stature) among basketball players than in other athletes. Similarly, the ECHO findings cannot be applicable to the general population, as well as to recreational basketball players, as the physical exertion cannot be compared among these groups. It is also important to acknowledge that our probands entered the study voluntarily and thus there might be selection bias, as players with perceived health problems may be more willing to participate.

## Conclusion

Echocardiographic abnormalities were observed in a minority of young basketball players and were mostly consistent with physiological “athlete’s heart”. Genetic findings were rare, but targeted genetic testing should be considered in selected individuals with dysmorphic features, suspicious echocardiographic findings, or a positive family history. Our findings support targeted screening of high-risk players, such as those who exhibit (mild) dysmorphic features or have a positive family history, including echocardiography. Early detection of cardiovascular risk can prevent life-threatening complications and enable safer athletic activity, particularly given the higher risk of mortality from heart disease that has been observed in professional basketball players.

## Data Availability

The original contributions presented in the study are included in the article/**Supplementary Material**. Further inquiries can be directed to the corresponding author/s.
