# Supplementary material for: Integrated cardiac, endocrine, and genetic assessment in adolescent basketball players
Source: Front Endocrinol (Lausanne). 2026 Jul 20;17:1851858. doi: 10.3389/fendo.2026.1851858 (PMC13429449; doi:10.3389/fendo.2026.1851858)
Supplement: Supplementary file 1 [file Table1.docx]

| **Phenotypic features** | **Number of points: 0** | **Number of points: 1** | **Number of points: 2** |
| --- | --- | --- | --- |
| Craniofacial | None | mild anomalies (e.g. broad nasal bridge) | marked features (e.g. dolichocephaly, macrocephaly, hypertelorism, frontal bossing) |
| Orofacial | None | mild (e.g. high-arched palate) | major dysmorphism (e.g. cleft palate, orofacial stigmatization) |
| Skeletal | None | mild scoliosis, genua valga, clinodactyly, pedes calcaneovalgi | pectus deformity, arachnodactyly, severe scoliosis |
| Skin / Connective tissue | None | single feature (e.g. striae, mild hyperelasticity) | > 2 features (e.g. striae + joint hypermobility/skin hyperlaxity) |
| Eye | None | myopia <3 dioptres, astigmatism, amblyopia, strabism | myopia > 3 diptres, ptosis, epicanthus, lens dislocation, >2 minor findings |
| Other | None | isolated finding (e.g. disproportionality, hallux, 1 café-au lait spot, fetal finger pads) | multiple minor findings (e.g. > 1 café-au-lait spots), hypotonia, macrosomia, gynoid habitus |

**Supplementary Table 1. Scoring system of dysmorphic features**

## Total score calculation and classification: 0 points → no dysmorphic features; 1–3 points → mild dysmorphic features; ≥4 points → clinically relevant dysmorphic features

| **Supplementary Table 2. Comprehensive characteristics of the cohort** | | | | | | | | | | | | | | | | | |
| --- | --- | --- | --- | --- | --- | --- | --- | --- | --- | --- | --- | --- | --- | --- | --- | --- | --- |
| **Proband number** | **Gender** | **Age (years)** | **Height (SD)** | **MPH (SD)** | **Birth weight (SD)** | **Birth length (SD)** | **SHSI (SD)** | **Medical history** | **Dysmorphic features** | **Dysmorphic severity** | **Suggestive phenotype** | **Gene** | **Associated syndrome** | **Nucleotide level *** | **ACMG** | **Positive family history for cardiovascular disease** | **ECHO finding** |
| Basketball players with tall stature (height >2 SD) | | | | | | | | | | | | | | | | | |
| 1 | F | 20 | 2.9 | 1.39 | 0.07 | 0.08 | -1.8 | Myopia (-4/-4 D) | Positive thumb sign, mild scoliosis, striae | Dysmorphic | Marfanoid | *SHANK3* | - | c.3736C>T | VUS | yes | - |
| 2 | F | 20 | 2.5 | 1.39 | 1.86 | 1.44 | -2.8 | - | Positive wrist sign, chest asymmetry, mild scoliosis, striae, clinodactyly | Dysmorphic | Marfanoid | *-* | - | - | - | yes | - |
| 3 | F | 16 | 2.5 | 0.59 | 1.43 | 0.53 | -2.51 | - | Positive wrist sign, striae, clinodactyly, higher palate | Dysmorphic | Marfanoid | *FBN1* | Marfan syndrome | c.3410G>A | VUS | yes | - |
| 4 | F | 17 | 3.1 | 2.90 | 1.63 | 1.89 | 3.8 | Myopia  (-1/-1.5 D) | Positive wrist sign, high- arched palate | Dysmorphic | Marfanoid | - | - | - | - | yes | Left atrium enlargement, physiological adaptation, follow up recommended |
| 5 | F | 18 | 3.1 | 1.92 | 2.22 | 1.89 | -2.2 | - | Clinodactyly, striae | Mildly dysmorphic | - | - | - | - | - | yes | - |
| 6 | F | 19 | 2.6 | 1.07 | 1.87 | 0.08 | 1.2 | Myopia  (-2.75/-2.5D) | Striae, mild scoliosis | Mildly dysmorphic | - | - | - | - | - | yes | - |
| 7 | F | 16 | 2.9 | 1.47 | 0.74 | 0.53 | -2.1 | Myopia (-1.25/-1.5D) | Striae, positive wrist sign, brachydactyly | Dysmorphic | - | *COL1A1* | - | c.2420C>A | VUS | yes | - |
| 8 | F | 14 | 2.4 | 2.98 | 1.89 | 1.59 | -0.4 | Myopia (-1/-1D) | Positive wrist and thumb sign, mild scoliosis, chest asymmetry | Dysmorphic | Marfanoid | *-* | - | - | - | yes | - |
| 9 | F | 16 | 2.4 | 1.61 | 2.14 | 1.44 | -2.0 | Myopia (-0.75/-0.75 D) | Thumb sign positive | Dysmorphic | - | *-* | - | - | - | yes | - |
| 10 | F | 19 | 3.5 | 0.59 | 0.17 | -0.38 | -2.1 | - | Pedes calcaneovalgi, brachydactyly, hypotonia, joint hypermobility | Dysmorphic | - | - | - | - | - | no | - |
| 11 | F | 15 | 3.5 | 2.13 | 2.11 | 1.89 | -2.4 | - | Positive thumb sign, higher palate, café au-lait spots, clinodaktyly,arachnodaktyly, macrocefaly | Dysmorphic | Marfanoid | - | - | - | - | no | - |
| 12 | F | 18 | 2.7 | 2.42 | 1.48 | 1.89 | -2.8 | - | disproportionality | Mildly dysmorphic | - | - | - | - | - | no | - |
| 13 | F | 16 | 3.8 | 1.78 | -0.89 | -1.28 | -0.1 | - | - | - | - | - | - | - | - | yes | - |
| 14 | F | 17 | 2.3 | 2.02 | 1.54 | 0.08 | -0.2 | - | Wrist and thumb sign positive, high- arched palate | Mildly dysmorphic | Marfanoid | *LRP4* | - | c.3052C>T | VUS | yes | - |
| 15 | F | 15 | 2.1 | 2.34 | 1.63 | 0.53 | -0.6 | Postinfectious glomerulonefritis | - | - | - | *-* | - | - | - | yes | Left ventricle hypertrophy, left atrium enlargement, athletic heart, physiological adaptation |
| 16 | M | 15 | 3.4 | 0.43 | -0.45 | -0.24 | -0.1 | Myopia (-2.5/-2.5D) | Positive thumb and wrist sign, scoliosis | Dysmorphic | Marfanoid | *TGFB3* | - | c.865G>A | VUS | yes | - |
| 17 | M | 18 | 2.5 | 0.67 | 1.72 | 1.11 | -1.2 | - | striae | Mildly dysmorphic | - | *LRP4* | - | c.5357T>C | VUS | no | - |
| 18 | M | 20 | 2.4 | 0.53 | 2.29 | -0.24 | -1.6 | - | striae | Mildly dysmorphic | - | *CHD8* | - | c.3380G>T | VUS | no | - |
| 19 | M | 18 | 3.3 | 1.82 | 1.72 | 1.11 | -2.6 | - | Wrist sign positive, mild scoliosis, high-arched palate, skin hyperlaxity | Dysmorphic | Marfanoid | - | - | - | - | no | - |
| 20 | M | 18 | 2.6 | 2.47 | 2.58 | 3.66 | 0.5 | - | - | - | - | - | - | - | - | no | Left atrium enlargement, left ventricle hypertrophy, athletic heart, physiological adaptation |
| 21 | M | 18 | 2.3 | 1.59 | 2.39 | 1.59 | -0.4 | - | Positive wrist sign | Mildly dysmorphic | - | - | - | - | - | yes | - |
| 22 | M | 16 | 3.5 | 0.53 | -1.04 | -0.67 | -1.3 | - | Pectus excavatum, striae | Mildy dysmorphic | Marfanoid | *FBN1* | - | c.7339G>A | P | no | - |
| 23 | M | 20 | 2.9 | 2.25 | -1.47 | 1.11 | -1.0 | - | Thumb sign positive, skin hyperlaxity | Dysmorphic | - | - | - | - | - | no | - |
| 24 | M | 15 | 3.5 | 1.68 | 0.5 | -0.24 | -0.1 | hyperhomocystenemia | - | - | - | - | - | - | - | yes | - |
| 25 | M | 20 | 3.1 | -0.26 | -0.45 | -0.24 | -1.2 | - | Dolihocephaly, mild scoliosis, skin hyperlaxity, striae | Dysmorphic | Marfanoid | - | - | - | - | no | - |
| 26 | M | 13 | 2.9 | 0.96 | -1.66 | -0.24 | -0.6 | - | - | - | - | *LRP4* | - | c.1541-2A>G | VUS | no | - |
| 27 | M | 13 | 2.4 | 0.89 | 1.53 | 0.65 | -0.5 | AITD | - | - | - | - | - | - | - | yes | - |
| 28 | M | 19 | 2.2 | 1.25 | 0.29 | -0.67 | -1.5 | Myopia (-1/-1,75 D) | Thumb sign positive, joint hypermobility, striae, mild scoliosis, pectus carinatum, flat foot | Dysmorphic | Marfanoid | - | - | - | - | no | - |
| 29 | M | 17 | 2.5 | 0.67 | -0.45 | -0.24 | 0 | - | Mild scoliosis | Mildly dysmorphic | - | - | - | - | - | yes | - |
| 30 | F | 15 | 3.2 | 0.76 | 2.09 | 2.08 | -3.4 | Sacralization of sixth lumbar vertebrae | Dolichocephaly, joint hypermobility, positive thumb and wrist sign, mild facial stigmatization | Dysmorphic | Marfanoid | *-* | - | - | - | yes | dilatation of the aortic root and ascendent aorta, borderline finding |
| 31 | F | 17 | 2.7 | -0.5 | 1.04 | 1.11 | -2.0 | FoA, patellar dyysplasia | Patellar dysplasia, dolichocephaly | Mildly dysmorphic | - | - | - | - | - | no | - |
| 32 | F | 16 | 2.2 | -0.54 | 1.7 | 1.11 | 0.2 | Myopia (-0.75D/-0.75 D) | Brachycephaly, striae, flat foot | Dysmorphic | - | - | - | - | - | no | - |
| 33 | F | 18 | 2.3 | -0.71 | 3.08 | 0.19 | -3.2 | - | Mild scoliosis | Mildly dysmorphic | - | - | - | - | - | no | - |
| Basketball players without tall stature (height <2 SD) | | | | | | | | | | | | | | | | | |
| 34 | F | 16 | 1.1 | 1.47 | 0.41 | 0.53 | -0.2 | Hemangioma | Thumb and wrist sign positive, striae, skin hyperlaxity, café au-lait spot | Dysmorphic | Marfanoid | - | - | - | - | yes | - |
| 35 | F | 15 | 1.1 | 0.85 | 0.41 | 0.53 | 0 | - | 3 cafe au-lait spots, striae | Mildly dysmorphic | - | - | - | - | - | yes | - |
| 36 | F | 17 | 0.6 | 1.53 | 1.06 | 0.98 | 0.7 | - | Mild scoliosis | Mildly dysmorphic | - | - | - | - | - | yes | Hypertrophy of left ventricle, dilatation of left atrium, athletic heart, physiological adaptation |
| 37 | F | 15 | 1.5 | 0.91 | 2.67 | 1.89 | -0.3 | Followed up for a murmour | Positive wrist sign | Mildly dysmorphic | - | - | - | - | - | no | Dilatation of ascendent aorta, mild mitral regurgitation, borderline finding |
| 38 | F | 15 | 0.7 | 0.59 | 1.06 | 0.08 | -0.5 | Myopia (-2.5 D/-2.75 D) | Positive wrist sign, mild scoliosis, striae | Dysmorphic | Marfanoid | *-* | - | - | - | no | - |
| 39 | F | 15 | 1.6 | 1.66 | 1.41 | 0.08 | -0.8 | Unsignificant valve regurgitation | Positive wrist sign, joint hypermobility, skin hyperlaxity | Dysmorphic | Marfanoid | - | - | - | - | no | - |
| 40 | F | 19 | 1.9 | 1.63 | 0.41 | 0.08 | -0.1 | - | Striae, scoliosis, skin hyperlaxity | Dysmorphic | - | - | - | - | - | no | - |
| 41 | F | 16 | 0.8 | 0.2 | -0.28 | -0.38 | 0.2 | Myopia -1.75 D/-1 D | Wrist sign positive, joint hypermobility, café au-lait spots | Dysmorphic | Marfanoid | *-* | - | - | - | yes | - |
| 42 | F | 16 | 0.3 | -0.49 | 1.23 | 0.53 | -0.7 | - | Joint hypermobility | Dysmorphic | - | - | - | - | - | no | - |
| 43 | F | 19 | 1.2 | 1.63 | 0.41 | 1.44 | -1.1 | Spontanneous pneumotorax | Dolichocephaly, striae | Mildly dysmorphic | - | *-* | - | - | - | no | - |
| 44 | M | 17 | 0.5 | 0.89 | 0.95 | -0.24 | -0.3 | 0 | 3 cafe au-lait spots | Mildly dysmorphic | - | - | - | - | - | yes | - |
| 45 | M | 17 | 1.4 | 1.61 | 0.13 | -0.67 | -0.9 | 0 | brachycephaly | Mildly dysmorphic | - | - | - | - | - | no | - |
| 46 | M | 16 | 1.6 | 0.32 | 0.59 | -0.67 | -1.4 | 0 | Positive thumb and wrist sign, joing hypermobility, striae, skin hyperlaxity | Dysmorphic | Marfanoid | CNEs of *SHOX* | - | chrX:674960-963680dup | VUS | no | Dilatation of left atrium, athletic heart, physiological adaptation |
| 47 | M | 16 | 1.9 | 1.75 | 1.23 | 2.11 | -1.6 | Myopia (-0.75 D/-1 D) | Positive wrist sign, scoliosis | Dysmorphic | - | - | - | - | - | yes | - |
| 48 | M | 17 | 1.9 | 1.89 | 0.82 | 0.19 | -1.3 | 0 | Positive wrist sign, chest excavation, skin hyperlaxity, striae, scoliosis | Dysmorphic | Marfanoid | - | - | - | - | no | - |
| 49 | F | 13 | 1.1 | 1.24 | 1.41 | 0.19 | -1.4 | FoA | 1 café au-lait spot | Mildly dysmorphic | - | - | - | - | - | yes |  |
| 50 | M | 16 | 0.9 | 1.89 | -0.45 | -0.24 | 0.3 | 0 | Dolichocephaly, flat foot, striae | Dysmorphic | *-* | *-* | - | - | - | yes | - |
| 51 | M | 13 | 0.5 | -0.54 | 1.02 | 0.65 | 0.4 | 0 | Hyperdolichocephaly, flat foot | Mildly dysmorphic | - | - | - | - | - | no | - |
| 52 | M | 19 | 0.1 | 0.1 | 1.47 | -0,24 | 0 | 0 | 0 | - | - | - | - | - | - | no | Left atrium enlargement, dilatation of ascendent aorta, borderline finding |
| 53 | M | 16 | 1.5 | 2.5 | 1.04 | 0.98 | 0.6 | 0 | Positive thumb sign, striae | Mildly dysmorphic | - | - | - | - | - | no | - |
| 54 | M | 19 | 0.9 | 0.53 | 1.06 | -0.24 | 0.7 | 0 | Striae, flat foot, mild scoliosis | Mildly dysmorphic | - | *DNMT3A* | - | - | VUS | no | - |
| 55 | M | 16 | 0.7 | 0.53 | -1.25 | -1.08 | -1.3 | Varicocele, myopia -2.5 D/2.75 D | Striae, joint hypermobility, skin hyperlaxity | Dysmorphic | Marfanoid | - | - | - | - | yes | potentially pathological hypertrophy of interventricular septum, potentially pathological finding |

Abbreviations: ACMG: American College of Medical Genetics and Genomics (ACMG) standards and guidelines; AITD: autoimmune thyroid disease; CNE: conserved non-coding elements; D: diopter; F: female; FoA: foramen ovale appertum; M: male; MPH: midparental height; P: pathogenic; SD: standard deviation; SHSI: Sitting height to subischial length ratio; VUS: variant of uncertain significance

Reference sequences: *CHD8:* NM_001170629.2*, COL1A1:* NM_000088.4*, DNMT3A*: NM_022552.5, *FBN1:* NM_000138.5*, LRP4:* NM_002334.4*, SHANK3:* NM_001372044.2*, TGFB3:* NM_003239.5., *SHOX:* hg19

**Supplementary Table 3. Echocardiologic findings**

| Participant number | Age | Sex | Height_ | Height_SD | BSA | IVS mm | IVS_z | LVID mm | LVID_z | LVPWd mm | LVPWd_z | LV_EF | ALA_z | E:A | E:E' | LA R strain % | RV_FAC | TAPSE | AOAN_z | AO_root mm | AO_root_z | AO_STJ_z | AO_ASC_z |
| --- | --- | --- | --- | --- | --- | --- | --- | --- | --- | --- | --- | --- | --- | --- | --- | --- | --- | --- | --- | --- | --- | --- | --- |
| 30 | 15 | F | 186 | 3.2 | 1.7 | 6.6 | -0.59 | 51.8 | 0.39 | 6 | -1.01 | 0.59 | 0.36 | 1.9 | 4.14 | 30 | 0.48 | 20.2 | 0.04 | 33.8 | 2.72 | 3.44 | 3.55 |
| 37 | 15 | F | 176 | 1.5 | 1.8 | 7.8 | 0.39 | 52.7 | 0.45 | 8.9 | 1.47 | 0.6 | 1.55 | 1.9 | 2.94 | 32 | 0.31 | 28.5 | 1.06 | 27.7 | 0 | -0.85 | 2.95 |
| 55 | 16 | M | 184 | 0.7 | 2.1 | 14.1 | 5.31 | 47.3 | -1.41 | 10.7 | 2.59 | 0.61 | 1.81 | 3 | 5.47 | 36 | 0.38 | 25.9 | 0.65 | 31.4 | 0.74 | 1.92 | 2 |
| 52 |  | M | 180 |  | 1.9 | 8.6 | 0.81 | 57.3 | 0.94 | 7.3 | -0.18 | 0.63 | 2.35 | 2.3 | 4.61 | 32 | 0.4 | 22.1 | 2.13 | 33.7 | 1.82 | 2.76 | 3.16 |
| 15 | 15 | F | 178 | 2.1 | 1.78 | 7.5 | 0.16 | 52.9 | 0.56 | 9.6 | 2.12 | 0.73 | 3.37 | 3.8 | 3.25 | 39 | 0.52 | 24.5 | 1.08 | 28.2 | 0.28 | 1.41 | 0.61 |
| 36 | 17 | F | 170 | 0.55 | 1.7 | 8 | 0.7 | 48 | -0.42 | 10.5 | 3.04 | 0.71 | 2.78 | 2.8 | 6.21 | 35 | 0.53 | 21.4 | -0.25 | 25.5 | -0.67 | -0.11 | -0.02 |
| 20 | 18 | M | 198 | 2.8 | 2.25 | 12.4 | 3.51 | 58.8 | 0.57 | 9.8 | 1.53 | 0.69 | 2.56 | 1.9 | 6.59 | 30 | 0.41 | 27 | 1.09 | 31.5 | 0.21 | 1.39 | -0.54 |
| 46 | 16 | M | 189 | 1.6 | 2.1 | 9.4 | 1.26 | 62 | 1.55 | 9.6 | 1.53 | 0.62 | 2.51 | 3.8 | 4.43 | 30 | 0.42 | 30.1 | 1.83 | 28.1 | -0.77 | 0.16 | 0.65 |
| 4 | 17 | F | 186 | 3.12 | 2.01 | 7.3 | -0.32 | 61 | 1.69 | 8.3 | 0.63 | 0.67 | 2.73 | 1.9 | 3.83 | 32 | 0.42 | 21.7 | 0.84 | 26.8 | -0.97 | -0.93 | 1.59 |

Abbreviations: IVS mm Interventricular septum thickness at end-diastole in mm; IVS_z Interventricular septum thickness at end-diastole (Z score); LVID mm Left ventricular internal dimension at end-diastole in mm; LVID_z Left ventricular internal dimension at end-diastole (Z score); ); LVPWd mm Left ventricular posterior wall thickness at end-diastole in mm; LVPWd_z Left ventricular posterior wall thickness at end-diastole (Z score); LV_EF Left ventricle ejection fraction; ALA_z Area of left atrium (Z score); E:A Ratio of E to A; E:E' Ratio of E to E'; LA R strain % Left atrium reservoir strain rate in % RV_FAC Right ventricle fractional area change; TAPSE Tricuspid Annular Plane Systolic Excursion; AOAN_z Aortic valve anulus (Z score); AO_root_mm Diameter of the aortic root in the sinuses of Valsalva in mm; AO_root_z Diameter of the aortic root in the sinuses of Valsalva (Z score); AO_STJ_z Diameter of the aortic root in ST junction (Z score); AO_ASC_z Diameter of the ascending aorta (Z score)
